# Design and Evaluation of Clove Oil-Based Self-Emulsifying Drug Delivery Systems for Improving the Oral Bioavailability of Neratinib Maleate

**DOI:** 10.3390/pharmaceutics16081087

**Published:** 2024-08-19

**Authors:** Radhika Rajiv Mahajan, Punna Rao Ravi, Riya Kamlesh Marathe, Ajay Gorakh Dongare, Apoorva Vinayak Prabhu, Łukasz Szeleszczuk

**Affiliations:** 1 Department of Pharmacy, Birla Institute of Technology and Science Pilani, Hyderabad Campus, Jawahar Nagar, Kapra Mandal, Medchal District, Hyderabad 500078, Telangana, India; p20200469@hyderabad.bits-pilani.ac.in (R.R.M.); h20211460240h@alumni.bits-pilani.ac.in (R.K.M.); h20211460249h@alumni.bits-pilani.ac.in (A.G.D.); h20211460252h@alumni.bits-pilani.ac.in (A.V.P.); 2 Department of Organic and Physical Chemistry, Faculty of Pharmacy, Medical University of Warsaw, Banacha 1 Str., 02-093 Warsaw, Poland; lukasz.szeleszczuk@wum.edu.pl

**Keywords:** low oral bioavailability, self-emulsifying drug delivery systems, ternary phase diagram, emulsification time, oral pharmacokinetics

## Abstract

Neratinib maleate (NM), a tyrosine kinase inhibitor, is used in the treatment of breast cancer. NM is orally administered at a high dose of 290 mg due to its low solubility and poor dissolution rate at pH > 3, as well as gut-wall metabolism limiting its bioavailability. Self-emulsifying drug delivery systems (SEDDSs) of NM were developed in the current study to improve its oral bioavailability. The oily vehicle (clove oil) was selected based on the solubility of NM, while the surfactant and the cosurfactant were selected based on the turbidimetric analysis. Three different sets were screened for surfactant selection in the preparation of SEDDS formulations, the first set containing Cremophor^®^ EL alone as the surfactant, the second set containing a mixture of Cremophor^®^ EL (surfactant) and Caproyl^®^ PGMC (cosurfactant), and the third set containing a mixture of Cremophor^®^ EL (surfactant) and Capmul^®^ MCM C8 (cosurfactant). Propylene glycol was used as the cosolubilizer in the preparation of SEDDSs. A series of studies, including the construction of ternary phase diagrams to determine the zone of emulsification, thermodynamic stability studies (involving dilution studies, freeze-thaw, and heating–cooling studies), turbidimetric analysis, and physicochemical characterization studies were conducted to identify the two most stable combinations of SEDDSs. The two optimized SEDDS formulations, TP16 and TP25, consisted of clove oil (45% *w*/*w*) and propylene glycol (5% *w*/*w*) in common but differed with respect to the surfactant or surfactant mixture in the formulations. TP16 was prepared using a mixture of Cremophor^®^ EL (surfactant) and Caproyl^®^ PGMC (cosurfactant) in a 4:1 ratio (50% *w*/*w*), while TP25 contained only Cremophor^®^ EL (50% *w*/*w*). The mean globule sizes were 239.8 ± 77.8 nm and 204.8 ± 2.4 nm for TP16 and TP25, respectively, with an emulsification time of <12 s for both formulations. In vitro drug dissolution studies performed at different pH conditions (3.0, 4.5, 6.8) have confirmed the increase in solubility and dissolution rate of the drug by TP16 and TP25 at all pH conditions compared to plain NM. An oral pharmacokinetic study in female Wistar rats showed that the relative bioavailability (Frel) values of TP16 and TP25 over the plain NM were 2.18 (*p* < 0.05) and 2.24 (*p* < 0.01), respectively.

## 1. Introduction

A new class of drugs that bind irreversibly to the adenosine triphosphate binding pocket of the human epidermal growth factor receptor (HER) has emerged in recent years. Epidermal growth factor receptors (EGFR, HER1, or ErbB1), HER2 (ErbB2, neu), HER3 (ErbB3), and HER4 (ErbB4) are the four members of the HER (ErbB) receptor tyrosine kinase family. Numerous cancer types have been linked to the overexpression, mutation, or abnormal activity of these receptors. Neratinib maleate (NM) is a type of tyrosine kinase inhibitor that is used for the treatment of breast cancer. It is also known as a pan-HER inhibitor due to its ability to bind to three of the four mentioned HER receptors, except for HER 3 [1]. NM is marketed under the brand name “Nerlynx” and has a high oral dose of 290 mg because of its low and variable oral bioavailability (BA) of 11–39%. In clinical settings, NM is administered through the oral route as six mini tablets with a total dose of 290 mg. The oral dose of NM is divided into six mini tablets to facilitate dose titration in patients experiencing dose-dependent gastrointestinal side effects such as severe diarrhea. The oral dose of NM is decreased by reducing one tablet (out of the initial six tablets at the start of therapy) at a time until the patient can cope with gastrointestinal side effects. At less than 120 mg, i.e., three tablets, discontinuation of therapy is recommended. Since NM is recommended as chronic therapy for one year, the dose-dependent side effects make it difficult to continue the medication due to patient discomfort, thereby resulting in noncompliance with the therapy [2,3]. Reducing the oral dose of NM by improving its oral absorption can alleviate the dose-dependent side effects of NM, increase patient comfort, and improve compliance with the therapy.

NM is a highly lipophilic drug with a Log P of 4.7. It exhibits pH-dependent solubility in the pH range of 1.2 to 7.4. It has good solubility under low pH conditions, with a saturation solubility (C_s_) of 100 ± 1 and 22 ± 0.5 mg/mL at pH 1.2 and 3.5, respectively. The solubility of NM decreases significantly as the pH increases. The drug is insoluble at pH 6 (C_s_ = 90 μg/mL) and above. The low and variable oral BA of NM is primarily due to its poor solubility in intestinal pH conditions and extensive CYP3A4-related metabolism in the gut wall during its intestinal absorption process. To address the problems associated with the available conventional therapy, researchers have designed a few modified or novel drug delivery systems. Fadilah S.A. et al. developed neratinib-loaded polyamidoamine dendrimer nanocapsules coated with trastuzumab to target breast cancer cells. The researchers could increase the drug concentration at the site of action and decrease its cytotoxicity with the nanocapsules compared to the conventional formulation of neratinib [4]. Mohamed R. et al. designed effervescent floating tablets of neratinib to enhance the solubility and residence time of the drug in the upper GIT and, thereby, the oral bioavailability [5]. Our research group has made efforts to enhance the oral BA of NM and thereby reduce the dose and dose-dependent side effects using lipid-based drug delivery systems (LBDDS). To date, no LBDDS has been explored for the treatment of breast cancer with NM. The current research aims to improve the solubility, dissolution rate, and oral absorption properties of NM.

Self-emulsifying drug delivery systems (SEDDSs) are LBDDS that are capable of dissolving and delivering large doses of lipophilic drugs in solubilized form. A SEDDS contains oil, a surfactant, and a cosurfactant or cosolubilizer. Several approved marketed formulations showcase the translational capabilities of SEDDSs which include Agenerase^®^ (amprenavir) by Glaxo Group, United Kingdom; Depakene^®^ (valproic acid) by AbbVie Inc., IL, USA; Rocaltrol^®^ (calcitriol) by Validus Pharmaceuticals, NJ, USA; Vesanoid^®^ (tretinoin) and Accutane^®^ (isotretinoin) by Roche, Basel, Switzerland; and Aptivus^®^ (tipranavir) by Boehringer Ingelheim, Rhein, Germany, etc. [6,7,8,9]. These systems rapidly form an emulsion upon dilution with water [10]. Suppose the sizes of the globules formed by the SEDDSs in the emulsion are >1 µm or between 0.1 µm and 0.8 µm. In that case, the SEDDSs are classified as self-microemulsifying drug delivery systems (SMEDDS) or self-nanoemulsifying drug delivery systems (SNEDDS), respectively. During the digestion process, gastric lipase in the stomach partially digests lipid components, whereas pancreatic lipase in the small intestine completes the digestion of lipids. The bile salts and cholesterol secreted as a part of the bile secretions through the biliary duct into the duodenum help in the micellar solubilization of the lipophilic drug. These micelles prevent drug precipitation and keep the drug in a solubilized form for the entire course of absorption in the gastrointestinal tract (GIT). The SEDDS formulations are reported to enhance the solubility, dissolution rate, and BA of lipophilic drugs such as atorvastatin, naringenin, cefpodoxime, and talinolol [11,12,13,14].

The objective of the current research was to design and optimize NM-loaded SEDDSs to improve the oral BA of the drug. The components used in the formulations were finalized scientifically. The NM-loaded SEDDSs were characterized for physicochemical properties, in vitro drug release, and stability to identify the optimized formulation with the desired characteristics. Finally, oral pharmacokinetic studies were conducted in Wistar rats to determine the efficacy of the optimized NM-loaded SEDDSs over the conventional formulation of NM in improving the BA of the drug.

## 2. Materials and Methods

### 2.1. Materials

Neratinib maleate (NM) and Rufinamide (internal standard in the bioanalytical method) were obtained from MSN Laboratories Pvt. Ltd. (Hyderabad, India) and Dr. Reddy’s laboratories (Hyderabad, India), respectively. Excipients like Gelucire^®^ 59/14 (Mixture of Lauroyl Polyoxyl-32 glycerides and PEG 6000), Gelucire^®^ 44/14 (Lauroyl polyoxyl-32 glycerides), Gelucire^®^ 48/16 (Polyoxyl-32 stearate (type I)), and Lauroglycol™ FCC (Propylene glycol monolaurate (type I, monoesters > 45%)), and solubilizing solvents such as Transcutol^®^ (diethylene glycol monoethyl ether) and Labrasol^®^ (Caprylocaproyl macrogol-8 glycerides) were generously gifted by Gattefosse (Mumbai, India). Capmul^®^ MCM C8 (propylene glycol monolaurate) and Capryol^®^ PGMC (Propylene Glycol Mono and Dicaprylate) were gift samples from Abitec corporation (Columbus, OH, USA). Additionally, BASF provided kind support by providing Cremophor^®^ EL (Polyethoxylated castor oil). A variety of essential oils were purchased from Naturalis (Leiden, The Netherlands), RM Herbals (Chennai, India), and Natures Natural (Ghaziabad, India). Clove oil and glacial acetic acid were procured from Sisco Research Laboratories, Pvt. Ltd. (Mumbai, India). Citric acid, sodium chloride (NaCl), and various buffer salts were obtained from SD Fine Chemicals Pvt. Ltd. (Mumbai, India). HPLC grade solvents acetonitrile (ACN) and methanol (MeOH) were purchased from Qualigens Pharma Pvt. Ltd. (Mumbai, India). Filtered Milli-Q water was obtained from our institute’s central Millipore (Millipore^®^, Bedford, MA, USA) unit facility. Female albino Wistar rats were procured from Vyas Biosciences (Hyderabad, India). Isoflurane (anesthesia) was obtained from UMA Enterprises (Hyderabad, India).

### 2.2. Selection of Oil and Cosolubilizer

The saturation solubility of NM was checked in various natural oils (lemon oil, citronella oil, soybean oil, and clove oil) by gradually adding NM to the oil and mixing thoroughly. The additions were continued until no solubility was observed. The mixtures were kept under observation overnight. Based on visual observations after 24 h, oils with a clear appearance and maximum solubility of NM were finalized, and only for the selected oils was a quantitative analysis performed using an HPLC-UV method. A similar process was followed for screening the most suitable cosolubilizer among the various cosolubilizers, such as propylene glycol, octanoic acid, Labrasol^®^, and Transcutol^®^.

### 2.3. Selection of Surfactant (S)

Surfactants such as Gelucire^®^ 44/14, Gelucire^®^ 59/14, Gelucire^®^ 48/16, and Cremophor EL were screened with the help of turbidimetric analysis. All the surfactants used in this study had HLB values greater than 11. The clove oil (selected from the screening of oils) and the individual surfactants to be screened were mixed at a ratio of 1:1. The isotropic mixture was slightly heated to a temperature of 37 °C, and 0.01 g of the mixture was then diluted to 10 mL (1000×) in 15 mL falcon tubes. The tubes were then fixed on a ROTOSPIN (Electrolab, Mumbai, India) and inverted at 50 rpm for 2 min. Samples were collected at 12, 24, 36, 48, 60, and 120 s of inversion movement, representing 10, 20, 30, 40, 50, and 100 rotations, respectively. The samples were kept undisturbed for 2 h and then analyzed using a UV spectrophotometer to determine the absorbance values of each sample at 650 nm. The transmittance of each sample was determined from its absorbance value using the following equation (Equation (1)). The ease of emulsion formation was noted by the number of rotations required to achieve turbidity [12,14].

(1) T (%)=antilog (2−a)

where ‘*T* (%)’ is the percentage transmittance and ‘*a*’ is the absorbance.

### 2.4. Selection of Surfactant and Cosurfactant Mixture (S_mix_)

Cosurfactants such as Lauroglycol™ FCC, Capryol^®^ PGMC, and Capmul^®^ MCM C8 were screened via turbidimetric analysis. The cosurfactants used in this study had HLB values ranging between 5 and 6. The surfactant (selected from the screening of surfactants) and the individual cosurfactants to be screened were mixed at a ratio of 2:1. The analysis was performed to check the emulsification ability of the surfactant with the cosurfactant, wherein the surfactant mixture has a combined HLB of surfactant and cosurfactant. The isotropic mixture containing the oil and surfactant mixture was homogenously formulated at a ratio of 1:1 and was slightly heated to a temperature of 37 °C. Then, 0.01 g of the mixture was diluted to 10 mL (1000×) in 15 mL falcon tubes. The tubes were then fixed on a ROTOSPIN (Electrolab, Mumbai, India) and inverted at 50 rpm for 2 min. Samples were collected at 12, 24, 36, 48, 60, and 120 s of inversion movement, representing 10, 20, 30, 40, 50 and 100 rotations, respectively. The samples were kept undisturbed for 2 h and then analyzed using a UV spectrophotometer to determine the absorbance values of each sample at 650 nm. The transmittance of each sample was determined from its absorbance value using Equation (1). The ease of emulsion formation was noted by the number of rotations required to achieve turbidity. As the ratio of cosurfactants to surfactants is the same in all the samples, the turbidity of the resulting nanoemulsions was used to assess the relative efficacy of each cosurfactant in forming the stable nanoemulsions [12,14].

### 2.5. Construction of Ternary Phase Diagrams

To determine the zone of emulsification, ternary phase diagrams of the formulations with different compositions of oil, surfactant alone, or a mixture of surfactant and cosurfactants (S_mix_), and cosolubilizer were plotted. Pouton’s classification was used to determine the percentage ranges of oil, S/S_mix_, and cosolubilizer for the SEDDSs [15]. The concentration of the oil varied between 35 and 50% *w*/*w*, the concentration of S/S_mix_ varied between 40 and 60% *w*/*w*, and the concentration of the cosolubilizer varied between 5 and 10% *w*/*w*. In all the formulations, the total percentage of the components (oil, S/S_mix_, and cosolubilizer) was set at 100%. Pseudo-ternary phase diagrams were constructed without incorporating the drug in the formulations. All the formulations prepared using different combinations of ingredients and at different percentages were evaluated for the emulsification time (ET) and the quality of the emulsion. The chemical compositions of the formulations are listed in Table 1, and the percentages of all three components used in the formulations are listed in Table 2.

To determine the ET, each formulation was prepared independently based on the compositions given in Table 2 to form a homogenous system. A clear glass vial containing 10 mL of filtered Milli-Q water was placed on a magnetic stirrer, maintained at 37 °C and 200 rpm. An aliquot of 200 µL of each formulation, preheated to 37 °C, was added to the vial. This represents a 50× dilution of the single dose of the formulation, mimicking the likely dilution that would be experienced by the SEDDS formulation in the gastric environment when administered through the oral route. The ET was assessed by determining the time taken for the formulation to form a homogenous emulsion following the dilution with water.

**Figure 1:**
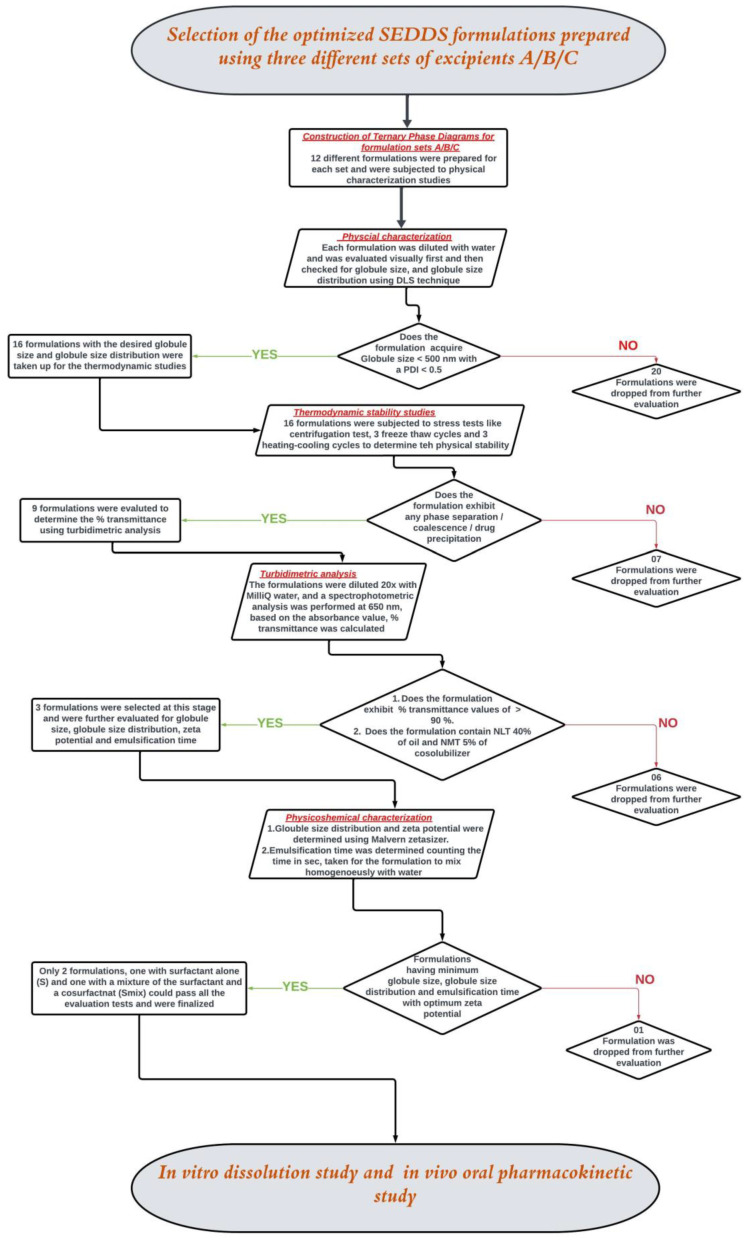
Decision tree for the selection of the optimized SEDDS formulations.

Further, samples were collected from the homogenous emulsions formed by the formulations upon dilution with water and analyzed for globule size (GS) and globule size distribution (GSD) using a Zetasizer (NanoZS, Malvern Instruments, Worcestershire, UK). The homogenous emulsions were then kept on a magnetic stirrer for 2 h at 100 rpm to check the possibility of phase separation or drug precipitation (in the case of drug-loaded formulations) to evaluate the physical stability of the emulsion-formed formulations. Based on the ET, GS, GSD, and stability for 2 h, the most suitable formulations were selected. Ternary phase diagrams were constructed separately for each set of ingredients. Each ternary phase diagram was constructed using 12 different formulations prepared by changing the proportions of the corresponding ingredients in that set. All the analyses were performed in triplicates. The zone of emulsification was then highlighted in each ternary phase diagram, using the best formulations that were selected based on the parameters mentioned in Figure 1 [12,14,16].

### 2.6. Physical Characterization of NM-Loaded SEDDSs

NM was loaded into SEDDS formulations identified from the zone of emulsification in each of the ternary phase diagrams. The NM-loaded SEDDSs were evaluated further step by step, as mentioned in the decision tree presented in Figure 1. The experimental procedures for the evaluations are described in the following sections (Section 2.6.1, Section 2.6.2 and Section 2.6.3).

#### 2.6.1. Thermodynamic Stability Test

Studies on thermodynamic stability, including centrifugation, heating–cooling cycles, and freeze–thaw cycles were conducted to assess phase separation, drug precipitation, and the impact of temperature fluctuations on SEDDS stability. To assess the stability of each formulation as an isotropic single-phase system, the formulation was diluted with water 20 times and centrifuged for 30 min at 3500 rpm. Further, the same set of formulations was incubated at 4 °C and 45 °C for 48 h in three heating–cooling cycles and three freeze–thaw cycles ranging between a freezing temperature of −20 °C and a thawing temperature of 25 ± 2 °C, i.e., controlled room temperature (CRT). Formulations that displayed any indication of phase separation, creaming or cracking, or drug precipitation in all the evaluations mentioned above were rejected. The stable formulations at all three thermodynamic conditions were only evaluated for the remaining studies [14].

#### 2.6.2. The % Transmittance

The NM-loaded SEDDSs were diluted with MilliQ water as the dispersion phase, and the resulting emulsions were visually examined for any signs of immiscibility of formulation with the dispersion phase. The experiments were carried out following a 20-fold dilution. MilliQ water was used as the blank scan before the samples were analyzed. A UV–vis spectrophotometer (Jasco, Easton, MD, USA) was used to measure the absorbance of the samples at 650 nm. The % transmittance was calculated using a mathematical equation by setting the absorbance values [14].

#### 2.6.3. Time for Self-Nano Emulsification

Using the bead and vial method, the time needed for specific formulations to self-emulsify was determined. A 20 mL transparent glass vial was filled with 10 mL of filtered Milli-Q water. The water was heated to 37 °C to mimic the physiological temperature. Using a micropipette, 200 μL of the chosen formulations were gradually added to the vial while stirring at 200 RPM on a magnetic stirrer. The ET was observed and recorded. Each study was repeated three times [14].

#### 2.6.4. Droplet Size, Polydispersity Index (PDI), and Zeta Potential Determination

Using differential laser scattering (DLS) technology, the average GS and GSD of the reconstituted formulations prepared in Section 2.6.3. were measured. The prepared emulsions were diluted 100 times with filtered MilliQ water before analysis. Three measurements were taken using a Zetasizer (NanoZS, Malvern Instruments, Worcestershire, UK). The principle of backscattering was applied, and the measuring angle was 173°. The intensity vs. particle size was used to obtain the values for the Z average and polydispersity index. Similarly, the zeta potential was also determined.

### 2.7. Composition of the Optimized NM-Loaded SEDDSs Selected Based on Physical Characterization Studies

The final compositions of the two selected formulations included 45% *w*/*w* clove oil, 50% Cremophor^®^ EL (S)/Cremophor^®^ EL: Capryol PGMC_4:1 (S_mix_), and 5% propylene glycol. To achieve a total dose of 290 mg, 2 g of clove oil was weighed accurately. It was heated to 70 ± 5 °C in a controlled setup. Once the desired temperature was reached, NM was added, and the mixture was stirred at a speed of 500 ± 50 RPM until a clear solution was obtained (30–45 s). The solution was then removed from the heating condition and allowed to cool before the addition of the remaining excipients. At room temperature, 2.22 g of S (TP25) or S_mix_ (TP16) was added and mixed correctly. This step was followed by the addition of a 0.22 g cosolubilizer. The final formulation was then stirred overnight at room temperature to attain homogeneity. After 24 h, the formulation was observed for any undissolved particles of the drug and stored in refrigerated conditions with airtight packing.

### 2.8. Stability Study

Samples of two different optimized formulation compositions were kept in a stability chamber (Remi, Mumbai, India) for three months in line with ICH guidelines. Three different conditions were maintained: a CRT of 25 ± 2 °C, a relative humidity (RH) of 60 ± 5%, a temperature of 40 ± 2 °C, an RH of 75 ± 5%, and refrigeration at 2–8 °C. Airtight 2 mL centrifuge tubes containing the samples were sealed and loaded into the respective chambers. Samples were collected every month for three consecutive months. At each time point, the assay and the physical appearance of the samples were evaluated. By comparing the data from the monthly sample collections with the freshly prepared formulation at the initiation of the stability study, the percent deviation was calculated. Every stable sample was tested in triplicate [16,17].

### 2.9. Preparation and HPLC Analysis of Assay Samples to Determine % Drug Loading (% DL)

High-performance liquid chromatography coupled with a photodiode array ultraviolet detector (HPLC-UV) was employed to analyze the samples from assay (DL (%) of formulations), in vitro dissolution, and stability studies. The samples were analyzed using a Phenomenex Kinetex C18 (250 mm length, 4.6 mm internal diameter, and 5 μm particle size) column in isocratic mode by pumping the mobile phase consisting of 60% aqueous phase (0.1% *v*/*v* orthophosphoric acid in water, pH adjusted to 2.5 ± 0.05) and 40% organic phase (MeOH) at a flow rate of 1 mL/min. The column was maintained at 25 °C, and the detection wavelength was fixed at 268 nm. The sample injection volume was 10 μL. The linearity, accuracy, and precision were determined for the developed HPLV-UV method. The method was linear in the calibration range of 1000–15,000 ng/mL, with a limit of quantification of 46 ng/mL.

To determine the assay of NM-loaded SEDDSs (for both freshly prepared formulations and stability samples), the formulations were slightly heated to 40 ± 2 °C under stirring at 500 rpm for 30 min (for viscosity reduction) to dispense the formulation for accurate weighing. In a 10 mL volumetric flask, 50 mg of the formulation was weighed accurately, followed by the addition of 5 mL of each MeOH and chloroform. The volumetric flask was closed airtight and vortex-mixed for 2 min. The volumetric flask was then bath-sonicated for 30 min to dissolve the drug completely in the mixture of solvents. The samples were taken from the mixture and diluted 100× before analysis using the above-mentioned HPLC-UV method.

### 2.10. In Vitro Dissolution Study

The accurately weighed mass of both the optimized final SEDDS formulations (equivalent to 10 mg of NM) was investigated for an in vitro drug dissolution study by dispersing the SEDDS mixture directly in 250 mL at pH 3.0 (citrate buffer), pH 4.5 (pH-adjusted ammonium acetate buffer), and pH 6.8 (pH-adjusted ammonium acetate buffer) as the dissolution medium. Type II (paddle type) dissolution apparatus was used to perform the experiment. The temperature of the media was maintained at 37 ± 0.5 °C with a stirring rate of 50 rpm. The TP16 and TP25 formulations were accurately weighed to a weight of 207 ± 0.5 mg (equivalent to 10 mg of NM) in triplicate. Dissolution studies of Plain NM (10 mg) were also conducted to compare the data with the two SEDDS formulations. At predetermined time intervals of 0, 15, 30, 45, and 60 min, samples (2 mL) were removed from the dissolution vessels and stored appropriately. Immediately after the sample collection, at each sampling time point, 2 mL of the respective fresh media (kept at 37 ± 0.5 °C) was replaced into the dissolution vessel. The collected samples were diluted 5 times with the mobile phase (0.1% orthophosphoric acid and MeOH in 6:4 ratio) and analyzed using the HPLC-UV method described in Section 2.9. Triplicate samples were used for in vitro drug dissolution investigations. The dissolution data of plain NM with TP16 and TP25 and between TP16 and TP25, at different time points, were compared using a *t*-test at a 5% level of significance.

### 2.11. In Vivo Pharmacokinetic Study

Female Wistar rats weighing 200–220 g were used for the oral pharmacokinetic studies. The animals were quarantined for 7 days immediately after procurement and acclimatized for 7–10 days in our institute’s animal facility (Animal House Registration number 1912/PO/RE/S/16/CPCSEA). The animal housing was maintained at 22 ± 1 °C (room temperature) and 55 ± 10% relative humidity with a 12 h light/dark cycle. Food and water were provided to the rats ad libitum during the quarantine and acclimatization periods. The protocol (Protocol No.: BITS-HYD-IAEC-2023-18) was approved by the Institutional Animal Ethics Committee (IAEC). The animals fasted for 12 h with access to only water prior to drug administration. The water access was removed before the study was initiated the next day. The animals were weighed before the study and three animals (*n* = 3) of approximately the same weight were used for determining the oral pharmacokinetics of each treatment (plain NM, TP16, and TP 25). The protocol for water administration was as follows: 1 mL immediately after the dosing, 0.5 mL at the 1st h, and 0.25 mL at every time point for 9 h (3rd, 5th, 7th, and 9th h). The drug dose for oral administration was fixed at 10 mg/kg for all the treatments (plain NM, TP16, and TP 25). The accurate amount of a treatment dose to be administered was determined based on the weight of the animal. The weighed amount of each treatment was carefully filled in size 9 capsules and administered to the corresponding rats. The capsules were administered using a specialized oral gavage, which delivers the capsules directly into the stomach. The blood samples were collected (200 ± 50 µL) via retro-orbital plexus puncture at pre-dose 1, 2, 3, 4, 6, 8, 12, 15, 22, and 30 h. Blood was collected in centrifuge tubes containing 25 μL of anticoagulant solution (4.5% *w*/*v* EDTA solution; one-part EDTA solution to nine parts blood). The animals were provided with access to food from the 9th h of dosing the treatments. The samples were processed and analyzed using a validated HPLC-UV method [18,19]. The plasma drug concentration vs. time data were analyzed using Phoenix^®^ WinNonlin software (version 8.3.5.340, Certara Inc., Durham, NC, USA). The data were subjected to noncompartmental analysis (NCA) to determine the pharmacokinetic parameters from the plasma time course data obtained from the oral administration of NM. The pharmacokinetic parameters obtained for the two SEDDS formulations (TP16 and TP25) were compared separately against the plain NM using a *t*-test at a 5% level of significance. In addition, the parameters of TP16 and TP25 were also compared with each other using the *t*-test.

## 3. Results and Discussion

### 3.1. Selection of Oil and Cosolubilizer

The oils (lemon oil, citronella oil, soybean oil, and clove oil) selected for screening were reported to be safe for oral administration and capable of solubilizing similar classes of drugs with poor aqueous solubility [20,21,22]. In addition, due to the presence of free fatty acids in higher percentages, the oils used in this study were reported to exhibit a lower pH range, which is favorable for solubilizing the salt of a weakly basic drug like NM. Among the oils tested in this study, NM showed a higher solubility of 104 ± 1.23 mg/mL in clove oil (C_s_ = 104 ± 1.23 mg/mL). Therefore, clove oil was selected as the oily vehicle for the preparation of NM-loaded SEDDS formulations.

The solubility of NM was high in propylene glycol (C_s_ = 4.77 ± 0.04 mg/mL) compared to any other cosolubilizer used in this study. NM was found only to form a suspension and had exhibited very less solubility of <1 mg/mL in all the remaining cosolubilizers (octanoic acid, propylene glycol, Transcutol^®,^ and Labrasol^®^). Propylene glycol was selected as the cosolubilizer in the preparation of NM-loaded SEDDS formulations.

### 3.2. Selection of Surfactant and Cosurfactant

#### 3.2.1. Selection of Surfactant

Different grades of Gelucire^®^ were identified as suitable surfactants based on their HLB values and ability to inhibit CYP3A4 enzymes [23,24,25,26]. Gut wall metabolism, mediated by CYP3A4 enzymes, is reported to play a significant role in the poor oral BA of NM [2]. The details of different grades of Gelucire^®^, screened as surfactants, in this study are presented in Table S1. Out of the four different grades of Gelucire^®^ identified from the literature, Gelucire^®^ 48/16 was found to be immiscible with water and, therefore, not taken for further studies. The remaining three surfactants showed a % transmittance of more than 90%, which indicated that three surfactants were able to form stable emulsions with clove oil. The ET was also found to be less than 30 s for the three surfactants. Gelucire^®^ 44/14 was dropped from further studies as it showed a transmittance value of 92%, which was the lowest among the three surfactants tested in this study. Gelucire^®^ 59/14 and Cremophor^®^ EL were taken up for further studies and used in combination with cosurfactants to form stable emulsions with clove oil.

#### 3.2.2. Selection of Cosurfactant

A mixture of one hydrophilic and one hydrophobic surfactant, which yielded an optimal HLB value, was added in the final SEDDS formulation to ensure the formation of a stable emulsion with low ET [27,28,29,30]. Since NM is a BCS Class IV drug, hydrophobic surfactants (which were supposed to act as cosurfactants in the S_mix_), which have the property to inhibit CYP3A4 enzymes and promote the lymphatic uptake process, were selected. Table S2 lists the cosurfactants used in this study. To obtain an optimal HLB value, a ratio of 2:1 (2 parts of hydrophilic surfactant and 1 part of hydrophobic surfactant) was used, as described in Section 2.3. The hydrophobic surfactants, due to their lesser proportion in the mixture, are considered cosurfactants in the formulations. The HLB of the surfactant mixture was calculated using the following Equation (2) [31,32].

(2) $$HLB\;of\;the\;Surfactant\;Mixture=F_{S}\times {HLB}_{S}+F_{CoS}\times {HLB}_{CoS}$$

where ${HLB}_{S}$ = HLB of the surfactant; ${HLB}_{CoS}$ = HLB of the cosurfactant; $F_{S}$: fraction of surfactant used in the mixture, and $F_{CoS}$: fraction of cosurfactant used in the mixture.

The % transmittance values obtained for the S_mix_ combinations are presented in Table 3. Lauroglycol^TM^ FCC, at a ratio of 2:1, showed the lowest % transmittance with both surfactants and, therefore, was dropped from further studies. Formulations prepared using Gelucire^®^ 59/14 solidified at room temperature. Therefore, Gelucire^®^ 59/14 was also dropped from further studies. Both the remaining combinations of Cremophor EL with Capryol^®^ PGMC and Capmul^®^ MCM C8 were found to be the best-performing combinations of surfactant to cosurfactant at a ratio of 2:1. Therefore, Capryol^®^ PGMC and Capmul^®^ MCM C8 were selected as the cosurfactants.

The mixture of Cremophor^®^ EL + Capryol^®^ PGMC and the mixture of Cremophor^®^ EL + Capmul^®^ MCM C8 were optimized for their ratios to be used further as the mixture of the surfactant. The evaluation was conducted at two ratios of 3:1 and 4:1 in addition to the initially studied ratio of 2:1. Among the formulations prepared using different ratios to prepare a mixture of surfactants, Cremophor^®^ EL + Capryol^®^ PGMC in 4:1 ratio (HLB = 12.5; % transmittance = 94.86 ± 0.48%) and a mixture of Cremophor^®^ EL + Capmul^®^ MCM C8 in 4:1 ratio (HLB = 12.6; % transmittance = 92.86 ± 0.7%) showed the highest % transmittance values. Based on the results of given trials, formulations containing Cremophor^®^ EL alone, a mixture of Cremophor^®^ EL + Capryol^®^ PGMC (in the ratio of 4:1), and a mixture of Cremophor^®^ EL + Capmul^®^ MCM C8 (in the ratio of 4:1) as surfactants were taken ahead for further evaluations. Using the above three sets, the ternary phase diagrams were constructed to identify the zone of emulsification individually for each of the sets.

### 3.3. Construction of Ternary Phase Diagrams

**Figure 2:**
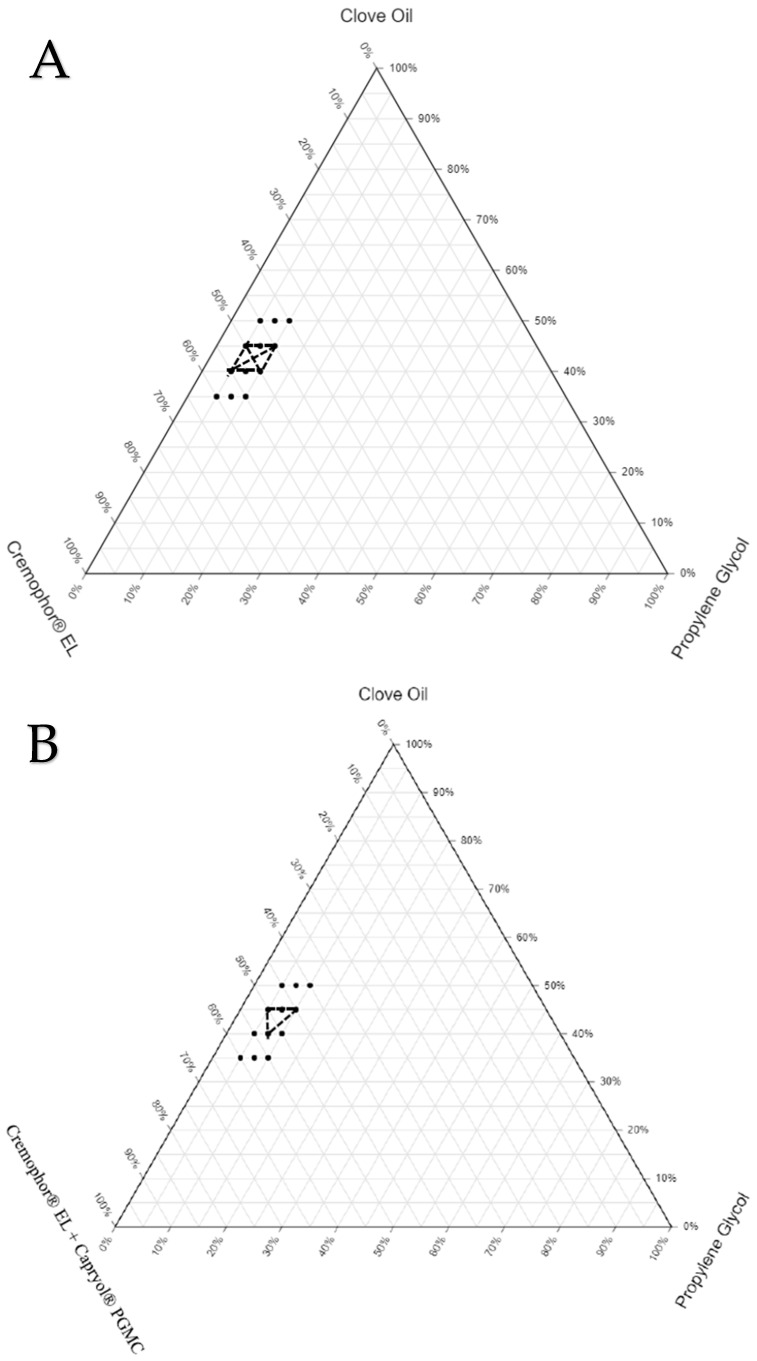

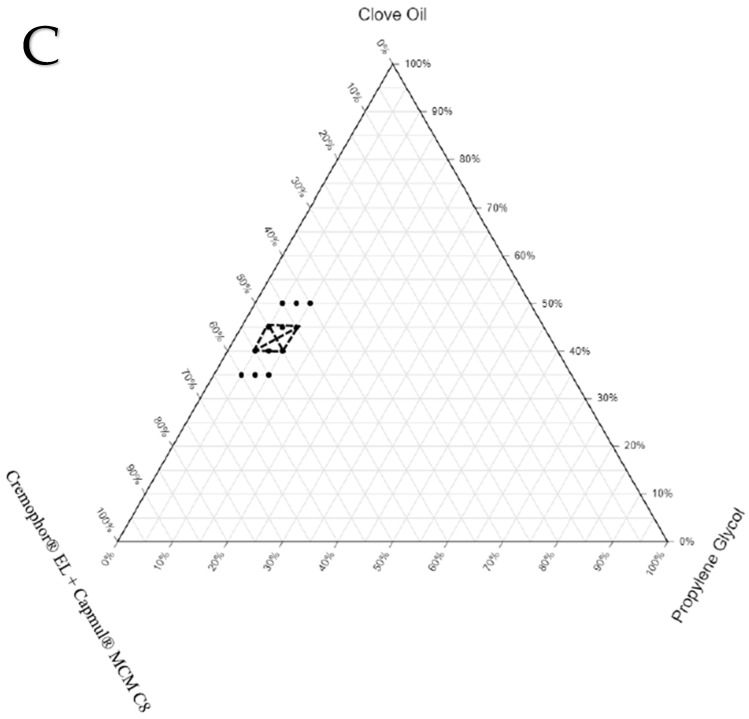
Ternary phase diagrams constructed for the three sets of SEDDS formulations: (**A**) SEDDSs set prepared using a mixture of clove oil + Cremophor^®^ EL + propylene glycol; (**B**) SEDDSs set prepared using a mixture of clove oil + Cremophor^®^ EL + Capryol^®^ PGMC + propylene glycol; (**C**) SEDDSs set prepared using a mixture of clove oil + Cremophor^®^ EL + Capmul^®^ MCM C8 + propylene glycol.

The final components selected for the three sets of SEDDS formulations were as follows: SEDDS set A—clove oil, Cremophor^®^ EL, and propylene glycol; SEDDS set B—clove oil, Cremophor^®^ EL + Capryol^®^ PGMC, and propylene glycol; SEDDS set C—clove oil, Cremophor^®^ EL + Capmul^®^ MCM C8, and propylene glycol. The ranges for the proportion of each excipient to be used in the preparation of the three sets of SEDDS formulations were identified based on a few preliminary trials. The drug was not completely soluble (at the required dose strength) when the proportion of oil was less than 35%. On the other hand, stable emulsion was not formed when the proportion of oil exceeded 50%. The range for the proportion of oil in all three sets of formulations was between 35% and 50%. The cosolubilizer proportion was kept minimal (5% to 10%) to avoid the possibility of drug precipitation in the gastrointestinal fluids due to the diffusion of the cosolubilizer from the formulation into the gastrointestinal fluids. Finally, the range for the proportion of S/S_mix_ was fixed between 40% and 60% to make the total proportion of all excipients in any given SEDDS formulations 100%. To locate the zone of emulsification for each set of SEDDS formulations, which provides an idea about the relationship between the formulation components and their ability to form a desired emulsion, ternary phase diagrams were constructed. The ternary phase diagrams constructed with the zone of emulsification are shown in Figure 2. The selected oil (clove oil), surfactant (Cremophor EL), cosurfactant (Capmul MCM C8 or Capryol^®^ PGMC), and cosolubilizer (propylene glycol) were varied within the predefined ranges. The total sum of all excipients used in all the SEDDS formulations was fixed at 100%. The formulation compositions are listed in Table 4.

To draw the zone of emulsification in each set, the boundary based on the compositions of SEDDSs, which yielded ET less than 15 s, GS less than 500 nm, and GSD less than 0.5 (following suitable dilution with water), was identified. The formulations prepared using the compositions both on the boundary and within the boundary were taken up for further characterization studies. In the ternary phase diagrams for each set, the boundary marked by dots shows the compositions of SEDDS formulations (36 formulations) screened for the selection of the zone of emulsification. The portion marked with dark solid lines represents the compositions of the actual zone of emulsification where the SEDDS formulations (16 formulations) yielded the desired properties upon dilution. These 16 formulations were loaded with the drug (NM) and subjected to thermodynamic stability studies via centrifugation, three freeze–thaw cycles, and three heating–cooling cycles.

### 3.4. Characterization of NM-Loaded SEDDS Formulations

#### 3.4.1. Thermodynamic Stability Test of 16 SEDDS Formulations

The stability of a formulation over a longer duration without compromising its physical appearance or drug content is the most crucial consideration when developing a formulation. SEDDS formulations can exhibit stability issues such as phase separation, drug precipitation, and drug degradation. These stability problems can occur during their storage or upon dilution with water (or gastric fluids following their oral administration). These factors can directly affect the safety and efficacy of the formulation. To evaluate the stability of SEDDSs, centrifugation (after 1:20 dilution with water) and temperature exposure studies were performed. The results of both studies are presented in Table 5.

Thermodynamic stability tests were performed to identify and eliminate unstable formulations. In the centrifugation test, each SEDDS formulation was diluted at a 1:20 ratio with water to mimic the worst-case scenario, i.e., when the gastric fluid content of the stomach was low. The formulation should be capable of emulsifying even at such low volumes of aqueous content and should keep the drug in the dissolved state. A total of seven formulations, TP6, TP7, TP8, TP9, TP17, TP18, and TP27, were eliminated after this study because of phase separation and the drug precipitation from the formulation. In the dilution test, the set of SEDDS formulations containing only Cremophor^®^ EL as the surfactant maintained their stability/homogeneity, even when the proportion of oil in the formulation was more than or equal to 45%. However, the set of SEDDS formulations containing the mixture of surfactants failed to maintain their stability/homogeneity when the proportion of oil in the formulation is more than or equal to 45%. This demonstrated the strong ability of Cremophor^®^ EL alone to form a stable and fine emulsion even in smaller volumes of aqueous fluid when the proportion of oil is high in the formulation. In total, nine formulations (TP4, TP5, TP14, TP16, TP22, TP23, TP24, TP25, and TP26) were found to be stable when subjected to three challenging thermodynamic stress tests.

#### 3.4.2. The % Transmittance of Nine Selected SEDDS Formulations

The % transmittance was measured to determine how well the S or S_mix_ helped the SEDDS formulation in the formation of a fine emulsion when mixed with the aqueous phase. In other words, we can assess the emulsification capacity of surfactants used in the preparation of SEDDSs. The higher the % transmittance of the SEDDS formulation (upon dilution with water), the better the emulsification capacity and the smaller the GS and GSD of the obtained emulsion. Table 6 presents the results obtained for the nine selected formulations from the thermodynamic stress studies. Values closer to 100% represent nanoemulsions as well as isotropic mixtures of components. TP4 and TP5 were dropped from further evaluation as their % transmittance values were less than 90%. The remaining seven formulations were further evaluated for GS, GSD, zeta potential, and emulsification time.

The % transmittance values obtained for the seven selected formulations mentioned in Table 6 did not have much variation, wherein all the values were found to be more than 91%. Since the drug solubility in all the sets of SEDDS formulations was affected by the proportion of oil, the formulations containing 45% or more of clove oil were considered to have a better ability to keep the drug in the dissolved state in the oil globules following the emulsification of the SEDDS formulations in the gastrointestinal fluids. In addition, the SEDDS formulations containing a lesser proportion of cosolubilizer (propylene glycol) would not suffer from issues of drug precipitation (particularly at higher dilutions) due to the diffusion of propylene glycol into the gastrointestinal fluids following the oral administration and emulsification of the formulation in the gastrointestinal tract. Based on the above two criteria, TP14, TP22, TP23, and TP24 were eliminated at this stage, and only three formulations (TP16, TP25, TP26) were taken ahead for the evaluating the ET, GS, GSD, and zeta potential.

#### 3.4.3. Emulsification Time of Three Selected SEDDS Formulations

All three selected formulations (TP16, TP25, and TP26) showed rapid emulsification with ET of less than 15 s. This suggests that Cremophor EL, both alone and in combination with CAPROYL^®^ PGMC, helps the designed SEDDS formulations to disperse quickly in aqueous media and rapidly form emulsions. Table 7 presents the ET values of the three formulations.

#### 3.4.4. Globule Size, Globule Size Distribution, Polydispersity Index, and Zeta Potential of Three Selected SEDDS Formulations

The GS and GSD represent the ability of the surfactant(s) (type and proportion of surfactant used in the SEDDSs) to form a fine and stable emulsion upon dilution with water. All the three formulations produced globules of nanometric size upon dilution with water. Formation of nanoemulsions by the SEDDS formulations can have a significant impact on the rate of drug partitioning from the globules into the gastrointestinal fluids and help in the overall increase in the oral absorption process of the drug. Table 7 shows the results for the three selected formulations.

TP16 was the only formulation from the set of SEDDS formulations prepared using the mixture of surfactants that could qualify all the evaluations; therefore, it was taken up for the final studies. In the set of SEDDS formulations prepared using Cremophor^®^ EL as the surfactant, though both TP25 and TP26 performed similarly in all the evaluations, TP25 was selected considering its lower GSD compared to TP26. The two final optimized formulations, TP16 (among the SEDDS formulations containing the mixture of surfactants) and TP25 (among the SEDDS formulations containing Cremophor^®^ EL as the surfactant), were further evaluated for stability studies, in vitro dissolution studies, and in vivo oral pharmacokinetic studies.

### 3.5. Validation Study for the GS, GSD, Zeta Potential, and ET of the Optimized SEDDS Formulations

The data presented in Table 7 show a high polydispersity index for the optimized SEDDS formulations (NM-loaded TP16 and TP25 formulations) that were characterized after they were subjected to various thermodynamic stress conditions. The data for NM-loaded TP16 and TP25 formulations presented in Table 7 do not correlate with the data of blank TP16 and TP25 (Table 4) with the same compositions evaluated to obtain the zone of emulsification in the ternary phase diagram. The higher polydispersity of the NM-loaded TP16 and TP25 compared to the blank TP16 and TP25 could be due to the exposure of the formulations to the various stress conditions. Therefore, to check the effect of drug loading on the physicochemical properties of NM-loaded TP16 and TP25 formulations, they were compared with the blank TP16 and TP25 formulations. Fresh batches of the NM-loaded TP16 and TP25-optimized SEDDS formulations were prepared, and the GS, GSD, zeta potential, and ET of the formulations were evaluated by following the same procedures as mentioned in Section 2.6.3 and Section 2.6.4. The results of the analysis are presented in Table 8.

### 3.6. Stability Study

**Figure 3:**
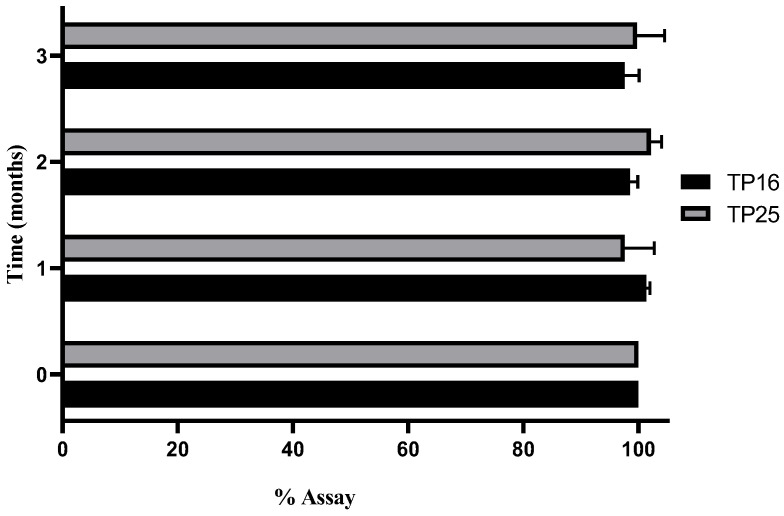
Assay (%) of TP16 and TP25 stored at refrigerated condition (2–8 °C).

Three different conditions were considered for stability evaluation. Out of the three conditions, CRT (25 ± 2 °C with RH of 60 ± 5%) and accelerated conditions (40 ± 2 °C with RH of 75 ± 5%) were found to be unsuitable for storage, for TP16 and TP25, as the assay dropped drastically below 80% for both the formulations. In refrigerated conditions (2–8 °C), both formulations were stable (in terms of drug assay, ET, GS, and GSD) for 90 days of stability study. The assay (%) of TP16 and TP25 for the samples collected at the end of each month over 3 months is shown in Figure 3.

Clove oil, which is one of the major constituents in both the optimized formulations, is volatile and prone to evaporation at temperatures above 25 °C. The decrease in the assay of the drug with time for the samples stored in CRT and accelerated conditions was primarily due to the loss of clove oil, which impacted the solubility of the drug in the SEDDS formulations. Ideally, the stability studies should have been conducted by encapsulating the formulations in soft gelatin capsules (or hard gelatin capsules suitable for liquid filling) to avoid such loss of clove oil and determine the true stability properties of the formulations at CRT and accelerated conditions.

### 3.7. In Vitro Dissolution Study

**Figure 4:**
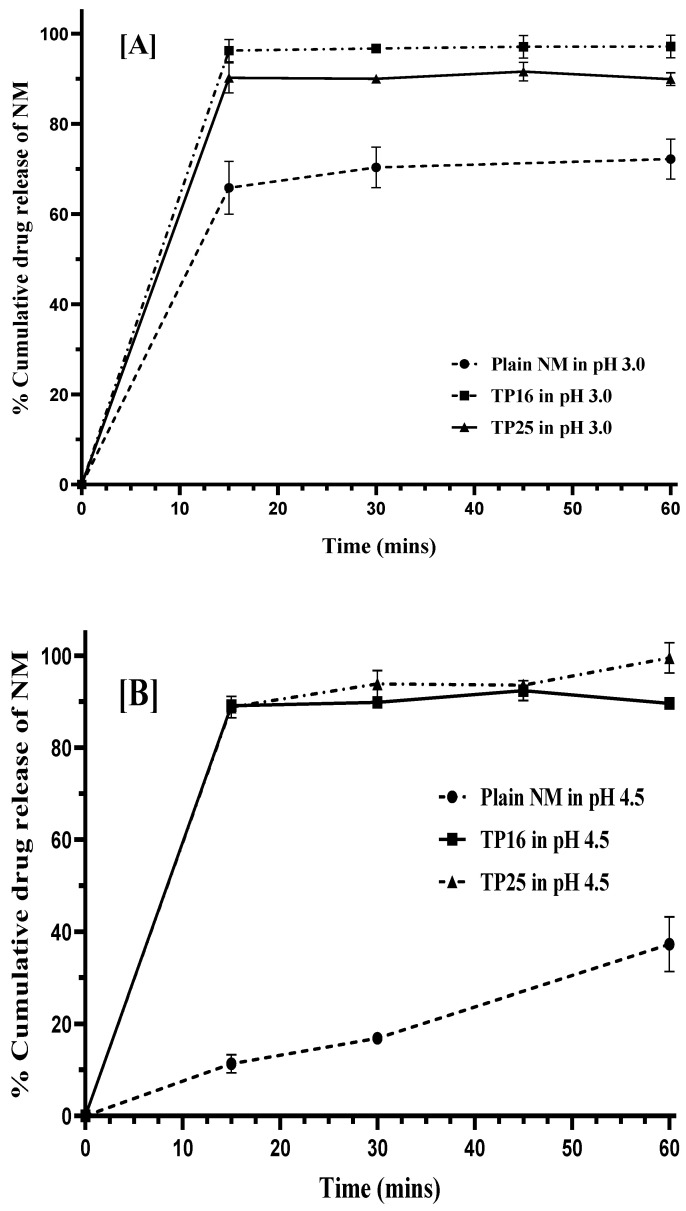

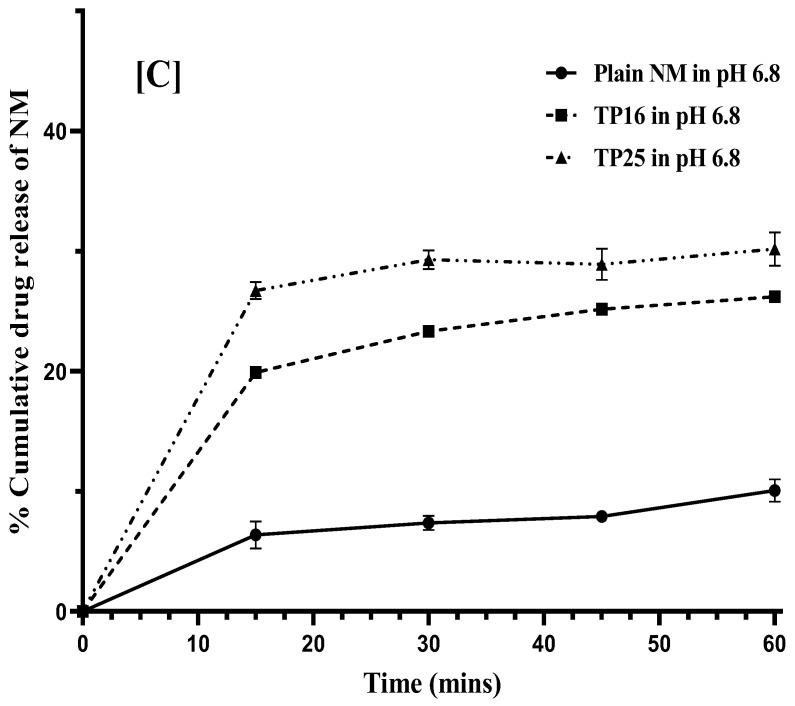
In vitro dissolution profiles of plain NM, TP16, and TP25. (**A**) pH 3.0, (**B**) pH 4.5, and (**C**) pH 6.8 Note: values represent the mean ± SD of *n* = 3 replicates.

NM exhibits good solubility below pH 3, while its solubility decreases sharply as the pH increases above 3. Therefore, in vitro dissolution studies were conducted in three different pH conditions: pH 3, pH 4.5, and pH 6.8. In vitro dissolution studies were performed in each of the three pH conditions without the use of any surfactant in the dissolution medium to evaluate the utility of the SEDDS formulations in improving the dissolution rate of the drug. In pH 3.0 dissolution medium, within 15 min, TP16 and TP25 showed rapid dissolution with 96.27 ± 2.28% (*p* < 0.01) and 90.23 ± 3.32% (*p* < 0.01) of drug dissolution compared to 65.84 ± 5.86% of drug dissolution for plain NM (Figure 4A). Similarly, plain NM (11.35 ± 1.99%) showed significantly lesser drug dissolution compared to TP16 (89.15 ± 0.75%) (*p* < 0.01) and TP25 (88.84 ± 2.32%) (*p* < 0.01) within 15 min in pH 4.5 (Figure 4B). In pH 6.8, TP16 and TP25 showed 19.90 ± 0.38% and 26.72 ± 7.72% of drug dissolution, respectively, at the end of 15 min., the drug dissolution was 3-times (*p* < 0.01) and 4-times (*p* < 0.01) lower for plain NM (6.38 ± 1.13%) compared to TP16 and TP25, respectively, in the same duration of this study (Figure 4C). The results obtained clearly indicate that both the SEDDS formulations (TP16 and TP25) could improve the solubility and dissolution rate of the drug compared to plain NM at all pH conditions. In the cases of TP16 and TP25, the drug dissolution could be due to the partitioning of the drug from the emulsion droplets formed by the SEDDS formulations following their addition to the dissolution media. The drug dissolution from both the SEDDS formulations was significantly higher due to the rapid ET and formation of emulsion with nanosized globules. This increased the surface area of contact between the emulsion droplets and the dissolution media, leading to rapid partitioning of the drug. In addition, the presence of surfactants in the SEDDS formulations could increase the affinity of the drug to the dissolution media. However, in the case of plain NM, due to the poor aqueous solubility, lack of any surfactant or solubilizer, and the surface area of the drug powder in contact with the dissolution media, the drug dissolution was comparatively lesser than that of SEDDS formulation.

In pH 6.8, TP25 exhibited significantly higher (*p* < 0.01) drug dissolution than TP16 at time points (15, 30, 45, and 60 min) during 1 h of this study. The difference in the dissolution data of TP25 and TP16 was more evident at pH 6.8 (where NM has the lowest solubility) than at pH 3 and pH 4.5. Statistically, no significant difference was observed in the drug dissolution of the two formulations till 1 h in pH 3 and pH 4.5. The importance of designing SEDDSs would be more apparent in the pH conditions where the drugs exhibit inherently low solubility. The difference in dissolution data of TP25 and TP16 in pH 6.8 could be due to the smaller GS and narrow GSD of the emulsion formed by TP25 compared to TP16. In addition, TP 25 had Cremophor^®^ EL at 50% proportion while TP16 had a mixture of Cremophor^®^ EL + CAPROYL^®^ PGMC in 4:1 ratio at 50% proportion in their respective formulations. The difference in the composition of surfactants used in both formulations could also be responsible for the differences in the drug dissolution process.

### 3.8. In Vivo Pharmacokinetic Study

**Figure 5:**
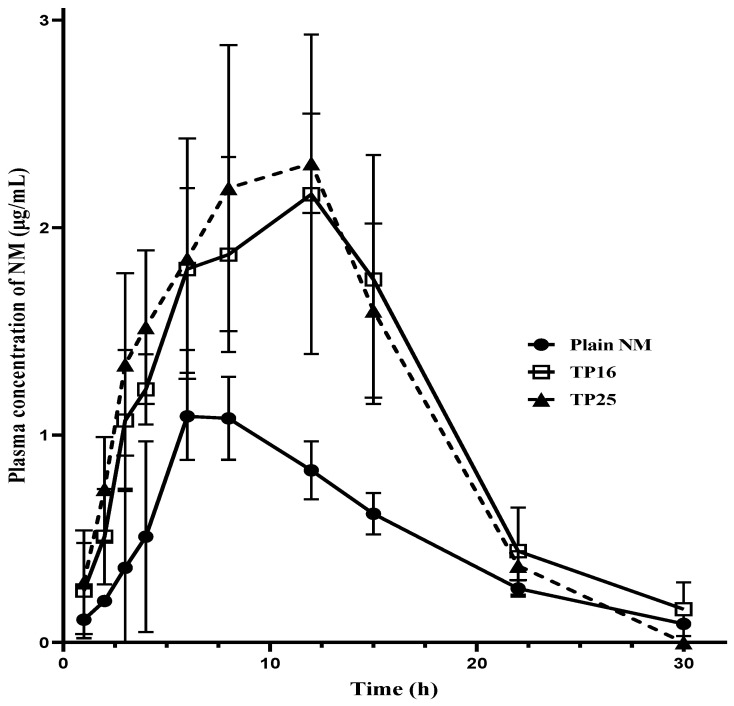
Oral pharmacokinetic profiles of Plain NM, TP16, and TP25 in female Wistar rats (*n* = 3) at a drug dose of 10 mg/Kg.

Blood samples obtained from oral pharmacokinetic studies were analyzed to construct the plasma concentration versus time profile for the three treatments (Figure 5). Each time point in the pharmacokinetic profiles of the three treatments (one for the plain NM and two for the SEDDS formulations) is a representation of the mean ± SD of independent samples from three female Wistar rats (*n* = 3). From the graph, it is evident that both the SEDDS formulations (TP16 and TP25) yielded higher C_max_ and AUC_last_ compared to plain NM. Although the C_max_ was delayed, the concentrations at the initial time points (including 1st, 2nd, 3rd, 4th, and 6th h time points) were prominently seen to be almost double for the SEDDS formulations than for the plain NM. This shows faster and higher plasma exposure of the drug from the SEDDS formulations compared to that from plain NM. The systemic exposure, represented in terms of the C_max_, increased by 1.97 times (*p* < 0.05) for TP16 and 2.30 times (*p* < 0.01) for TP25 compared to plain NM. Similarly, the AUC_last_ increased by 2.18 times (*p* < 0.05) and 2.24 times (*p* < 0.01) for TP16 and TP25, respectively, compared to plain NM. However, the rate and extent of absorption of NM from both TP16 and TP25 were found to be similar, with no significant difference in their C_max_, AUC_last,_ and MRT_last_ values.

The pharmacokinetic parameters are listed in Table 9. The relative bioavailability values of SEDDS formulations (TP16 and TP25) were determined by taking the ratio of the AUC_last_ of the SEDDS formulations to the AUC_last_ of the plain NM. Both the SEDDS formulations offered higher rates and extent of absorption of the drug compared to plain NM. Other research groups also witnessed the effect of suitable surfactants on the enhancement of oral bioavailability of specific drug candidates. Erica F. et al. demonstrated that Cremophor EL, well known for its property of enhancing intestinal absorption of drug molecules, helps increase the oral bioavailability of 4,6,40-Trimethylangelicin [33]. Further, with Cremophor EL again, the oral bioavailability of betulinic acid was found to increase by 15-fold [34].

The ingredients used in the SEDDS formulations are very critical towards the apparent difference in the absorption properties of the formulations compared to plain NM. The optimized combination of oil, S/S_mix,_ and cosolubilizer could have resulted in rapid dispersion of self-emulsifying systems in the gastrointestinal fluids, leading to enhanced solubility and the dissolution rate of the drug, particularly at pH > 3. Further, the ingredients in the SEDDSs could have formed micelles of the solubilized drug to prevent any precipitation at pH > 3, as well as in faster absorption of micelles (with the added surfactants) or mixed micelles (formed by the added surfactants in combination with endogenous bile acids) of the drug via lipid absorption pathways [35]. In addition, the nanoemulsion droplets formed by the SEDDS formulations in the gastrointestinal tract, particularly near the small intestine, could have been absorbed directly by pinocytosis [36]. Such an absorption process (nanoemulsion droplets by pinocytosis and micelles/mixed micelles by lipid absorption pathways) would minimize the chances of gut-wall metabolism (NM is a substrate for CYP3A4 enzymes in intestines) of NM during its transport across the gastrointestinal membranes and contribute to the overall increase in the bioavailability of the drug. Further, Cremophor^®^ EL, and Capryol^®^ PGMC, the surfactants used in the SEDDSs, are known to inhibit CYP3A4 enzymes [37,38,39]. These surfactants can reduce the chance of CYP3A4-mediated gut-wall metabolism of NM during the passive transport of molecules of NM (in a dissolved state in the luminal area) through the enterocytes. Overall, one or more of the different mechanisms could be involved in the increase in oral bioavailability of NM by the SEDDS formulations compared to plain NM. Both the optimized SEDDS formulations can reduce the oral dose of NM by at least half compared to plain NM (conventional formulations of NM), which will reduce the pill burden and, more importantly, dose-dependent gastrointestinal side effects.

## 4. Conclusions

Self-emulsifying drug delivery systems of NM were successfully optimized using clove oil as an oily vehicle, Cremophor^®^ EL alone as surfactant/mixture of Cremophor^®^ EL, and Capryol PGMC as a surfactant and propylene glycol as the cosolubilizer. The SEDDS formulations were optimized based on physical properties like emulsification time, % transmittance values, globule size, and globule size distribution. Ternary phase diagrams were constructed to identify the zone of emulsification. Thermodynamic stress tests like centrifugation, freeze-thaw, and heating–cooling cycles were performed on formulations that were part of the zone of emulsification. Formulations were further screened based on % transmittance, globule size, globule size distribution, zeta potential, and emulsification time of the formulations. Finally, two formulations, TP16 (containing a mixture of Cremophor^®^ EL and Capryol PGMC as surfactant) and TP25 (containing Cremophor^®^ EL alone as surfactant), were found to produce high % transmittance, low emulsification times, and nanosized globules with narrow globule size distribution. TP16 and TP25 formulations exhibited higher rates and extent of drug dissolution in pH 3.0, pH 4.5, and pH 6.8 dissolution media compared to plain NM in the in vitro dissolution studies. The relative oral bioavailability of NM increased two-fold from both the SEDDSs (TP16 and TP25) compared to plain NM. The optimized SEDDS formulations can reduce the oral dose of NM by 50%, which is clinically very significant considering the severe dose-dependent gastrointestinal side effects of the drug.

## Figures and Tables

**Table 1:** SEDDS compositions used to construct ternary phase diagrams.

Ternary Phase Diagram (TP)	Oil Phase	Surfactant	Cosurfactant	Cosolubilizer
A	Clove oil	Cremophor^®^ EL	Capmul^®^ MCM C8	Propylene glycol
B	Clove oil	Cremophor^®^ EL	Capryol^®^ PGMC	Propylene glycol
C	Clove oil	Cremophor^®^ EL	-	Propylene glycol

**Table 2:** List of formulations prepared for plotting the ternary phase diagrams.

Formulation Compositions			Oil (% *w*/*w*)	Surfactant/S_mix_ (% *w*/*w*)	Cosolubilizer (% *w*/*w*)
A	B	C			
TP1	TP10	TP19	35	60	5
TP2	TP11	TP20	35	57.5	7.5
TP3	TP12	TP21	35	55	10
TP4	TP13	TP22	40	55	5
TP5	TP14	TP23	40	52.5	7.5
TP6	TP15	TP24	40	50	10
TP7	TP16	TP25	45	50	5
TP8	TP17	TP26	45	47.5	7.5
TP9	TP18	TP27	45	45	10
TP31	TP28	TP34	50	45	5
TP32	TP29	TP35	50	42.5	7.5
TP33	TP30	TP36	50	40	10

**Table 3:** The % transmittance and emulsification times of formulations prepared using different surfactant mixtures (S_mix_).

Sr. No.	Surfactant Mixtures	Combined HLB	% Transmittance *	No. of Rotations
1	Gelucire^®^ 59/14 + Lauroglycol™ FCC	10.99	76.16 ± 0.48	10
2	Cremophor^®^ EL + Lauroglycol™ FCC	10.99	47.44 ± 0.95	10
3	Gelucire^®^ 59/14 + Capryol^®^ PGMC	11.32	93.86 ± 0.28	20
4	Cremophor^®^ EL + Capryol^®^ PGMC	11.32	87.54 ± 2.09	10
5	Gelucire^®^ 59/14 + Capmul^®^ MCM C8	11.49	93.27 ± 1.18	10
6	Cremophor^®^ EL + Capmul^®^ MCM C8	11.49	70.62 ± 0.99	10

* Data are presented as the means ± SDs of *n* = 3 replicates.

**Table 4:** Globule size, globule size distribution, and emulsification time of selected formulations from the pseudo-ternary phase diagrams of the three sets of SEDDS formulations.

Sr. No.	Composition of Surfactant	Formulation Trial No.	Z Average * (nm)	GSD * (PDI)	D10 * (nm)	D50 * (nm)	D90 * (nm)	Emulsification Time * (s)
1	Cremophor^®^ EL + Capmul^®^ MCM C8	TP4	336.77 ± 19.47	0.38 ± 0.03	266.33 ± 10.69	446.00 ± 22.52	609.33 ± 53.53	10.33 ± 1.53
2		TP5	385.28 ± 25.60	0.38 ± 0.24	341.67 ± 18.34	465.00 ± 22.65	694.67 ± 28.15	15.17 ± 1.26
3		TP6	365.33 ± 11.06	0.35 ± 0.04	270.70 ± 16.05	398.33 ± 50.33	486.33 ± 24.58	15.33 ± 1.53
4		TP7	385.90 ± 17.96	0.39 ± 0.03	285.00 ± 18.33	412.40 ± 38.33	580.00 ± 78.82	9.00 ± 1.00
5		TP8	260.13 ± 34.39	0.40 ± 0.06	75.73 ± 8.44	327.47 ± 31.31	509.67 ± 69.37	7.17 ± 0.76
6		TP9	376.13 ± 27.76	0.38 ± 0.02	283.83 ± 33.84	433.00 ± 28.16	576.00 ± 81.28	10.17 ± 0.76
7	Cremophor^®^ EL + Capryol^®^ PGMC	TP14	375.53 ± 24.42	0.45 ± 0.05	323.40 ± 24.16	504.67 ± 73.11	756.17 ± 67.25	10.50 ± 1.32
8		TP16	289.50 ± 1.10	0.42 ± 0.02	183.33 ± 14.19	304.67 ± 11.72	434.67 ± 20.03	12.17 ± 1.26
9		TP17	334.13 ± 29.29	0.36 ± 0.04	252.53 ± 49.21	379.10 ± 23.15	537.30 ± 54.96	12.83 ±1.04
10		TP18	284.07 ± 17.37	0.43 ± 0.06	64.13 ± 9.93	373.33 ± 26.69	524.67 ± 97.76	10.33 ± 1.04
11	Cremophor^®^ EL	TP22	333.53 ± 10.57	0.50 ± 0.02	253.67 ± 32.87	393.07 ± 23.77	553.33 ± 45.24	9.33 ± 1.53
12		TP23	309.33 ± 23.92	0.32 ± 0.03	320.33 ± 21.73	433.33 ± 20.03	535.00 ± 35.55	11.17 ± 1.26
13		TP24	362.93 ± 18.71	0.43 ± 0.03	362.73 ± 23.17	538.20 ± 35.50	749.43 ± 49.61	11.33 ± 0.58
14		TP25	345.53 ± 27.86	0.39 ± 0.01	345.73 ± 22.11	484.33 ± 29.02	670.63 ± 79.88	6.67 ± 0.25
15		TP26	394.80 ± 10.66	0.23 ± 0.04	336.33 ± 16.86	456.43 ± 38.71	642.07 ± 33.68	11.17 ± 0.76
16		TP27	334.87 ± 25.31	0.39 ± 0.06	194.00 ± 30.64	343.33 ± 39.55	434.43 ± 58.95	10.50 ± 1.80

* The data values are presented as the means ± SDs of three independent formulation samples.

**Table 5:** Results of thermodynamic studies, including centrifugation, freeze–thaw (F/T), and heating–cooling cycle (H/C) studies.

Sr. No.	Trial No.	Composition of the SEDDSs			Stability Tests			Inference
			Smix/S (%)	Cosolubilizer (%)	Centrifugation	F/T	H/C	
1	TP4	40	Cremophor^®^ EL+ CAPMUL^®^ MCM C8 in 4:1 ratio (55%)	5	√	√	√	PASS
2	TP5	40	Cremophor^®^ EL+ CAPMUL^®^ MCM C8 in 4:1 ratio (52.5%)	7.5	√	√	√	PASS
3	TP6	40	Cremophor^®^ EL+ CAPMUL^®^ MCM C8 in 4:1 ratio (50%)	10	×	√	√	FAIL
4	TP7	45	Cremophor^®^ EL+ CAPMUL^®^ MCM C8 in 4:1 ratio (50%)	5	×	√	√	FAIL
5	TP8	45	Cremophor^®^ EL+ CAPMUL^®^ MCM C8 in 4:1 ratio (47.5%)	7.5	×	√	√	FAIL
6	TP9	45	Cremophor^®^ EL+ CAPMUL^®^ MCM C8 in 4:1 ratio (45%)	10	×	√	√	FAIL
7	TP14	40	Cremophor^®^ EL + CAPROYL^®^ PGMC in 4:1 ratio (52.5%)	7.5	√	√	√	PASS
8	TP16	45	Cremophor^®^ EL + CAPROYL^®^ PGMC in 4:1 ratio (50%)	5	√	√	√	PASS
9	TP17	45	Cremophor^®^ EL + CAPROYL^®^ PGMC in 4:1 ratio (47.5%)	7.5	×	√	√	FAIL
10	TP18	45	Cremophor^®^ EL + CAPROYL^®^ PGMC in 4:1 ratio (45%)	10	×	√	√	FAIL
11	TP22	40	Cremophor^®^ EL (55%)	5	√	√	√	PASS
12	TP23	40	Cremophor^®^ EL (52.5%)	7.5	√	√	√	PASS
13	TP24	40	Cremophor^®^ EL (50%)	10	√	√	√	PASS
14	TP25	45	Cremophor^®^ EL (50%)	5	√	√	√	PASS
15	TP26	45	Cremophor^®^ EL (47.5%)	7.5	√	√	√	PASS
16	TP27	45	Cremophor^®^ EL (45%)	10	×	√	√	FAIL

**Table 6:** The % transmittance and self-emulsification time of the 9 selected formulations.

Sr. No.	Trial No.		Composition of the SEDDSs		% Transmittance *
		Oil (%)	S/S_mix_ (%)	Cosolubilizer (%)	
1	TP4	40	Cremophor^®^ EL + CAPMUL^®^ MCM C8 in 4:1 ratio (55%)	5	84.0 ± 0.2
2	TP5	40	Cremophor^®^ EL + CAPMUL^®^ MCM C8 in 4:1 ratio (52.5%)	7.5	87.5 ± 0.2
3	TP14	40	Cremophor^®^ EL + CAPROYL^®^ PGMC in 4:1 ratio (52.5%)	7.5	95.0 ± 0.1
4	TP16	45	Cremophor^®^ EL + CAPROYL^®^ PGMC in 4:1 ratio (50%)	5	90.9 ± 0.12
5	TP22	40	Cremophor^®^ EL (55%)	5	96.1 ± 0.1
6	TP23	40	Cremophor^®^ EL (52.5%)	7.5	96.1 ± 0.1
7	TP24	40	Cremophor^®^ EL (50%)	10	95.1 ± 0.1
8	TP25	45	Cremophor^®^ EL (50%)	5	92.7 ± 0.1
9	TP26	45	Cremophor^®^ EL (47.5%)	7.5	95.9 ± 0.1

* The values are presented as the means ± SDs of *n* = 3 replicates.

**Table 7:** Average globule size, globule size distribution, zeta potential, and self-emulsification time for selected formulations.

Sr. No.	Formulation Type	Trial No.	Z Average * (nm)	GSD (PDI) *	Zeta Potential * (mV)	Emulsification Time (s)
1	S_mix_	TP16	239.8 ± 77.8	0.8 ± 0.11	32.2 ± 2.3	<10
2	S	TP25	204.8 ± 2.4	0.6 ± 0.05	33.5 ± 1.2	<12
3	S	TP26	189.6 ± 43.6	0.6 ± 0.04	32.8 ± 4.4	<10

* Values represent the mean ± SD of *n* = 3 replicates.

**Table 8:** Validation of the physicochemical data obtained for the optimized SEDDS formulations (NM-loaded TP16 and TP25).

Sr. No.	Formulation Type	Trial No.	Z Average * (nm)	GSD (PDI) *	Zeta Potential * (mV)	Emulsification Time * (s)
1	S_mix_	TP16	274.75 ± 27.51	0.54 ± 0.12	32.56 ± 1.4	9.7 ± 1.04
2	S	TP25	289.69 ± 35.22	0.49 ± 0.10	34.12 ± 0.8	8.83 ± 0.76

* The values are presented as the means ± SDs of *n* = 3 replicates.

**Table 9:** Pharmacokinetic parameters obtained for plain NM, TP16, and TP25 administered through the oral route at a dose of 10 mg/Kg in female Wistar rats (*n* = 3).

Parameter	Plain NM ^a^	TP16 ^a^	TP25 ^a^
C_max_ (μg/mL)	1.11 ± 0.21	2.19 ± 0.72 *	2.55 ± 0.37 **^,#^
T_max_ (h) ^b^	6.0	12.0	12.0
AUC_last_ (h × μg/mL)	15.16 ± 0.97	33.12 ± 9.86 *	33.94 ± 4.24 **^,#^
AUC_inf_ (h × μg/mL)	15.83 ± 1.09	34.27 ± 10.42 *	34.08 ± 4.24 **^,#^
MRT_last_	11.74 ± 0.84	11.71 ± 0.60	10.99 ± 0.86 **^,#^
F_rel_	-	2.18	2.24

**^a^** The values are expressed as mean ± SD of *n* = 3 independent determinations except for the T_max,_ which is presented as the median; * statistically significant compared to the corresponding parameter of plain NM with *p* < 0.05; ** statistically significant compared to the corresponding parameter of plain NM with *p* < 0.01; ^#^ statistically insignificant compared to the corresponding parameter of TP16 with *p* ˃ 0.05. ^b^ The T_max_ values are represented as a median of *n* = 3 animals.

## Data Availability

The original contributions presented in this study are included in the article/Supplementary Material; further inquiries can be directed to the corresponding author.

## Supplementary Materials

The following supporting information can be downloaded at https://www.mdpi.com/article/10.3390/pharmaceutics16081087/s1, Table S1: list of shortlisted surfactants with HLB ≥ 11; Table S2: list of shortlisted cosurfactants with HLB ≤ 7.
