# Supplementary material for: Design and Evaluation of Clove Oil-Based Self-Emulsifying Drug Delivery Systems for Improving the Oral Bioavailability of Neratinib Maleate
Source: Pharmaceutics. 2024 Aug 19;16(8):1087. doi: 10.3390/pharmaceutics16081087 (PMC11358973; doi:10.3390/pharmaceutics16081087)
Supplement: Supplementary file 1 [file pharmaceutics-16-01087-s001.zip › pharmaceutics-3158671-supplementary.pdf]

Design and Evaluation of Clove Oil-Based Self-Emulsifying Drug Delivery Systems for Improving the Oral Bioavailability of Neratinib Maleate

Radhika R. Mahajan [1], Punna Rao Ravi [1]*, Riya Kamlesh Marathe [1], Ajay Gorakh Dongare [1], Apoorva V. Prabhu [1] and Łukasz Szeleszczuk [2]

1 Department of Pharmacy, Birla Institute of Technology and Science Pilani, Hyderabad Campus, Jawahar Nagar, Kapra Mandal, Medchal District, Hyderabad 500078, Telangana, India; p20200469@hyderabad.bits-pilani.ac.in (R.R.M.); h20211460240h@alumni.bits-pilani.ac.in (R.K.M.); h20211460249h@alumni.bits-pilani.ac.in (A.G.D.); h20211460252h@alumni.bits-pilani.ac.in (A.V.P.)
2 Department of Organic and Physical Chemistry, Faculty of Pharmacy, Medical University of Warsaw, Banacha 1 Str., 02-093 Warsaw, Poland; lukasz.szeleszczuk@wum.edu.pl
* Correspondence: rpunnarao@hyderabad.bits-pilani.ac.in; Tel.: +91-4066303539

SUPPLEMENTARY DATA

Table S1 List of shortlisted surfactants with HLB ≥11

Brand Name (Company name)	Chemical name	HLB	Bioavailability enhancer	% transmittance*	No. of rotations
Cremophor® EL (BASF)	Polyoxyl-35 castor oil, PEG-35 castor oil, Macrogolglycerol ricinoleate	14	Yes	97.67 ± 1.15	10
Gelucire® 44/14 (GATTE FOSSE)	Mono, di- and triglycerides and mainly PEG-32 (MW 1500) mono- and diesters of lauric acid (C12)	11	Yes	92.33 ± 1.53	30
Gelucire® 59/14 (GATTE FOSSE)	Lauroyl polyoxyl-32 glycerides Polyethylene Glycol 6000	14	Yes	95.33± 0.58	20
Gelucire® 48/16 (GATTE FOSSE)	Polyoxyl-32 stearate (type I) NF	12	Yes	Rejected based on less miscibility with water	

*Data represented as Mean ± SD for n=3 replicates.

Table S2 List of shortlisted cosurfactants with HLB ≤ 7

Brand Name	Company	Chemical name	HLB	Bioavailability enhancer
Lauroglycol™ FCC	GATTE FOSSE	Propylene glycol monolaurate (type I)	5	Yes
Capryol® PGMC	GATTE FOSSE	Propylene Glycol Mono and Dicaprylate NF	6	Yes
Capmul® MCM C8	ABITEC	Mono/Di Glycerides of Caprylic acid	6.5	Yes
